# Telomere Visualization in Tissue Sections using Pyrrole–Imidazole Polyamide Probes

**DOI:** 10.1038/srep29261

**Published:** 2016-07-06

**Authors:** Asuka Sasaki, Satoru Ide, Yusuke Kawamoto, Toshikazu Bando, Yukinori Murata, Mari Shimura, Kazuhiko Yamada, Akiyoshi Hirata, Kiyoshi Nokihara, Tatsumi Hirata, Hiroshi Sugiyama, Kazuhiro Maeshima

**Affiliations:** 1 Structural Biology Center, National Institute of Genetics, and Department of Genetics, Sokendai (Graduate University for Advanced Studies), Mishima, Shizuoka 411-8540, Japan; 2 Department of Chemistry, Graduate School of Science, Kyoto University, Sakyo, Kyoto 606-8502, Japan; 3 Department of Pathology, Hospital, National Center for Global Health and Medicine, Shinjuku, Tokyo 162-8655, Japan; 4 Department of Intractable Diseases, Research Institute, National Center for Global Health and Medicine, Shinjuku, Tokyo 162-8655, Japan; 5 Department of Surgery, Hospital, National Center for Global Health and Medicine, Shinjuku, Tokyo 162-8655, Japan; 6 HiPep Laboratories, Nakatsukasa-cho 486-46, Kamigyo-ku, Kyoto 602-8158, Japan; 7 Division of Brain Function, Department of Integrated Genetics, National Institute of Genetics, and Department of Genetics, Sokendai, Mishima, Shizuoka 411-8540, Japan

## Abstract

Pyrrole–Imidazole (PI) polyamides bind to specific DNA sequences in the minor groove with high affinity. Specific DNA labeling by PI polyamides does not require DNA denaturation with harsh treatments of heat and formamide and has the advantages of rapid and less disruptive processing. Previously, we developed tandem hairpin PI polyamide probes (TH59 series), which label telomeres in cultured cell lines more efficiently than conventional methods, such as fluorescence **in situ** hybridization (FISH). Here, we demonstrate that a TH59 derivative, HPTH59-b, along with immunostaining for specifying cell types in the tissues, visualizes telomeres in mouse and human tissue sections. Quantitative measurements of telomere length with single-cell resolution suggested shorter telomeres in the proliferating cell fractions of tumor than in non-tumor tissues. Thus, PI polyamides are a promising alternative for telomere labeling in clinical research, as well as in cell biology.

Mammalian telomeres consist of a long array of repetitive sequence (TTAGGG) and cap chromosome ends to prevent chromosome instability. Without the telomerase activity that can elongate telomeres, telomere length shortens with every cell division. Upon reaching a critical short length, the telomeres can trigger senescence, which suppresses the abnormal growth associated with tumorigenesis^1^,^2^,^3^. Telomerase reactivation can induce cells to circumvent growth limitation by telomere-dependent senescence and lead to immortalization^4^,^5^. Induction of the recombination between telomeres, termed alternative lengthening of telomeres (ALT), can also lead to a similar effect on the cells^6^,^7^.

Telomere length can be used as a diagnostic marker to detect immortalized cells with short telomeres or ALT cells with much longer telomeres. Thus far, quantitative fluorescent *in situ* hybridization (Q-FISH) with a peptide nucleic acid (PNA) probe has been widely used to visualize relative telomere length in individual cells^8^,^9^,^10^. Many clinical studies of telomere length measurement by Q-FISH have demonstrated that some types of cancer cells have shortened telomeres^11^,^12^,^13^. However, the FISH method requires harsh treatment using heat and 50% formamide for probe hybridization, which carries the risk of destroying cellular structures. Indeed, only a few studies have performed telomere labeling along with immunostaining for a cell marker (e.g., tumor marker) that can provide results with physiological relevance in human tissue sections^14^. Additionally, simple and less time-consuming steps and labeling reproducibility, such as clinical studies with numerous samples, are preferable for high-throughput experiments. The ‘gold standard’ for labeling telomeres, FISH, still has issues to be resolved, including for instance, the time required and convenience.

*N*-methylpyrrole (P)−*N*-methylimidazole (I) (PI) polyamides bind to the minor groove of duplex DNA without denaturation and can recognize Watson–Crick base pairs (Fig. 1A)^15^,^16^,^17^,^18^,^19^,^20^,^21^,^22^. PI polyamide is an alternative to the nucleic acid probes, which require a hybridization step to denature target DNA, for labeling specific DNA sites of the genome. Maeshima *et al*. reported on fluorescent tandem hairpin PI polyamide (TH59) probes that target human telomere sequences (TTAGGG)_n_ under mild conditions^23^. A hairpin structure is composed of two antiparallel linear compounds folded as a U-shape and is connected to another hairpin moiety through a hinge segment (Fig. 1B). Previously, to increase the binding selectivity of TH59, we developed a method for the robust synthesis of TH59 and various derivatives of TH59 with higher specificity for human telomeres in the cell^24^,^25^,^26^.

Here, we show that HPTH59-b, which has a hinge with an optimal length between two hairpins, visualizes telomeres not only in cultured cells but also in mouse and human frozen tissue sections (Fig. 1C). The tissue sections were co-stained with HPTH59-b and antibodies for cell-specific markers to compare the telomere lengths in various cell populations in the tissue sections. By quantitatively measuring telomeres in clinical tissue from an esophageal cancer patient, we found that a cell population positive for a proliferation marker, Ki-67 protein, has shorter telomeres than do the Ki-67-negative cells in non-tumor tissue sections. Therefore, PI polyamide provides an advantageous alternative for the measurement of telomere length in clinical research.

## Results

### Telomere length measurement by quantitative PI polyamide labeling

**Figure 2:**
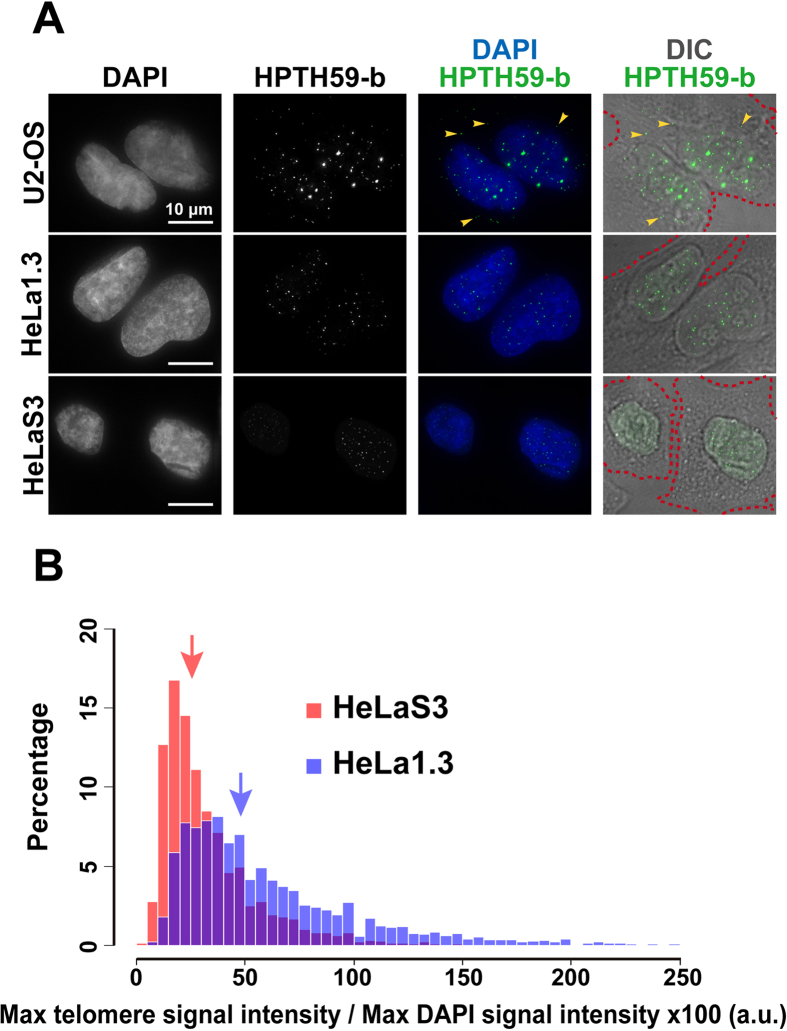
Quantitative telomere labeling using HPTH59-b. (**A**) Telomere labeling with HPTH59-b in human cultured cells. Telomerase-active cells (HeLa1.3 and HeLaS3) and alternative lengthening of telomere (ALT) cells (U2-OS) were stained with DAPI (first column) and HPTH59-b (second column). Merged images are in the third column. The fourth column shows merged images of HPTH59-b staining and differential interference contrast (DIC) images. Note that some U2-OS cells have extra-nuclear telomere signals (yellow arrowhead). (**B**) Distribution histograms of telomere signal intensities in HeLa1.3 (long telomere; in blue) and HeLaS3 (short telomere; in red) cells. The overlapping area of the two distributions is shown in purple. The numbers of telomere signals measured for HeLa1.3 and HeLaS3 cells are 13171 dots from 280 cells and 10805 dots from 277 cells, respectively. Median values of the intensity distributions for HeLa1.3 and HeLaS3 are 50.0 (blue arrow) and 26.7 (red arrow), respectively. To compare these median values, the Wilcoxon rank sum test was used (P < 0.01).

Several studies have shown that telomeres in cancer tissues are often shorter than those in normal tissues^27^,^28^,^29^; thus, quantitative telomere measurement should make an important contribution to investigations of tumorigenesis. To verify the quantification of telomere length by PI polyamide labeling, we stained the telomeres in cells having different lengths of telomere repeats with fluorescently labeled HPTH59-b^25^ and 4′, 6-diamidino-2-phenylindole (DAPI) (Fig. 2A,B). The telomere lengths of HeLaS3, HeLa1.3, and U2-OS ALT cells are 2–10 kb, ~23 kb, and <3 to >50 kb, respectively^30^,^31^.

As shown in Fig. 2A, HeLa1.3 cells exhibited more intense HPTH59-b signals than did HeLaS3 cells. Quantitative measurement of their signal intensities normalized to the DAPI signal indicated that the telomere signals of HeLa1.3 cells were significantly higher than those of HeLaS3 (Fig. 2B). Moreover, quantitative measurement of telomere signals on chromosomal spreads also showed that the signals at every chromosomal end of HeLa1.3 cells were higher than those of HeLaS3 cells (Supplementary Fig. S1A,B), suggesting that the telomeres of HeLa1.3 are longer than those of HeLaS3 cells. In U2-OS cells, we observed the strongest telomere signals among three cell lines (Fig. 2A). Additionally, U2-OS cells had several intense signals even outside the nucleus, which have been reported to be extra-chromosomal telomere DNA in the cytoplasm^32^. These results suggested that the observed signal intensity of HPTH59-b can distinguish different telomere lengths among these cultured cells.

### Telomere labeling in tissue sections

**Figure 3:**
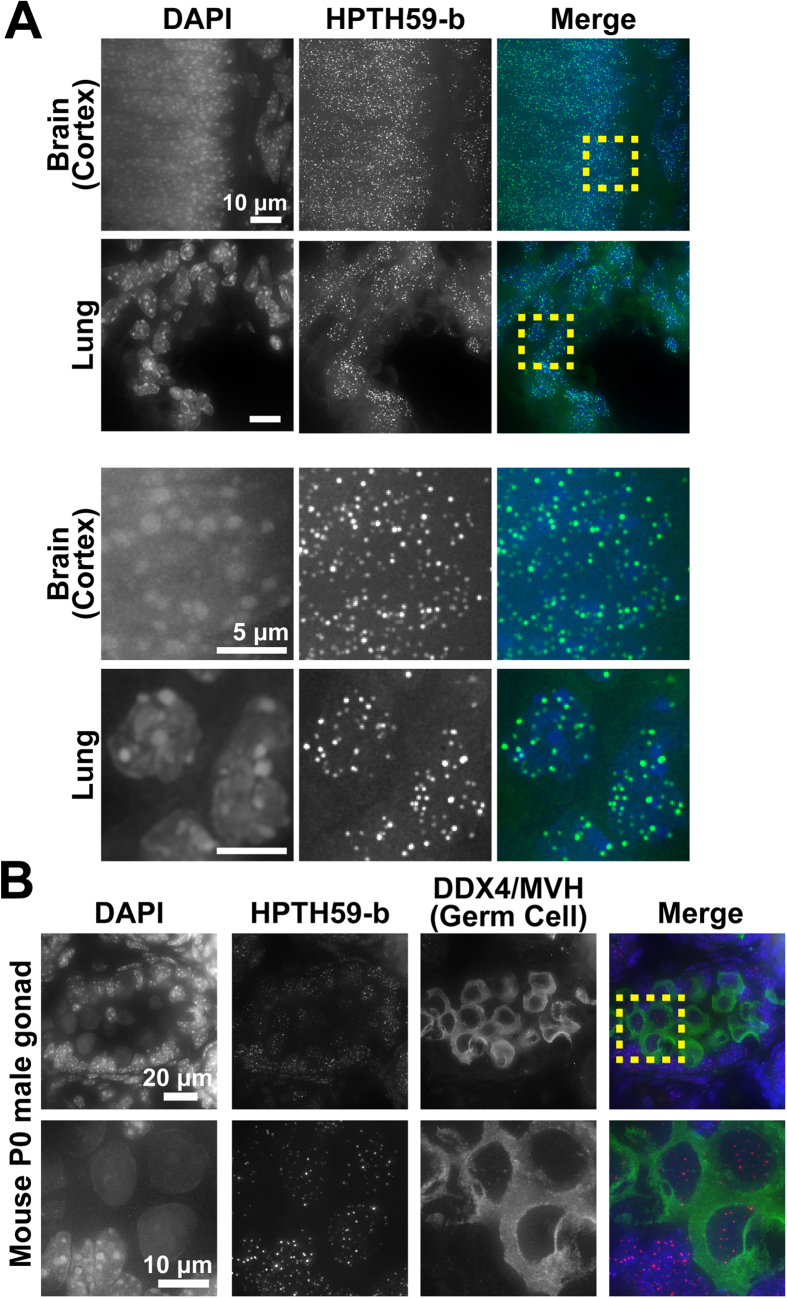
Telomere labeling in frozen mouse tissue sections. (**A**) First column, DAPI signal; second column, HPTH59-b signal; third column, merged images of DAPI and HPTH59-b. Enlarged images of the boxed region in the upper part are shown in the lower part. (**B**) Neonatal (P0) mouse gonads stained with DAPI (blue), anti DDX4/MVH antibody (germ cell marker; green), and HPTH59-b (red). Enlarged images of the boxed region in the first-row images are shown in the second row.

Next, to examine whether the telomere labeling by HPTH59-b can be applied to cells in tissues, we prepared frozen tissue sections of a mouse (20-μm thickness) and stained them with fluorescent HPTH59-b (green). Images of lung and brain regions are presented in Fig. 3A. Intense HPTH59-b foci in the DAPI-staining DNA region were clearly detected in both tissues. To test the telomere-targeting efficiency of HPTH59-b, we stained mouse tissue sections with HPTH59-b and anti-TRF1 (telomere-binding protein) antibody simultaneously and compared the signals. We observed strong signals of HPTH59-b with low background noise, whereas TRF1 signals were weak with high background noise in the brain and lung (Supplementary Fig. S2). Additionally, substantially more signals were detected by HPTH59-b than by TRF1 (Supplementary Fig. S2). On the other hand, in cultured murine cells (MC12), the intensity and number of HPTH59-b signals were comparable to those of TRF1signals (Supplementary Fig. S2). Thus, the HPTH59-b probe might label telomeres in tissue sections more efficiently than immunostaining.

In telomere biology, information on telomere length in a certain cell population in tissues, especially at the single-cell level, is very useful. To explore telomere length in a certain cell type, we focused on telomere length in a germ cell line, which has high telomerase activity. DEAD box proteins (DDX), putative RNA helicases, are specifically and highly expressed in the germ cell lineage in both sexes, and they are widely used as a marker of germ cell lineage^33^,^34^. Testis sections containing gonadal tissues, where primordial germ cells (PGCs) are located, were treated with HPTH59-b (red) and anti-DDX4/MVH antibody (green). Immunostaining with anti-DDX4/MVH antibody specifically labeled the cytosol of PGCs, and HPTH59-b showed clear foci in both the germ cells and somatic cells (Fig. 3B). The telomere signals in PGCs seemed to be weaker than those in PGC marker-negative cells. The PGC marker-positive cells showed a unique characteristic of nuclear organization: less DAPI staining over the nucleus, which is consistent with a previous report^35^. The quantitative measurement of telomere signals normalized to the DAPI signal suggested that PGCs had slightly longer telomeres than the PGC-negative cells (Supplementary Fig. S3). These results demonstrated that HPTH59-b highlighted telomeres and the antibody marked PGCs simultaneously, thereby demonstrating that double staining with HPTH59-b and antibodies can be used to label telomeres in a specific cell lineage in tissue.

### Shorter telomeres in human tumor cells

**Figure 4:**
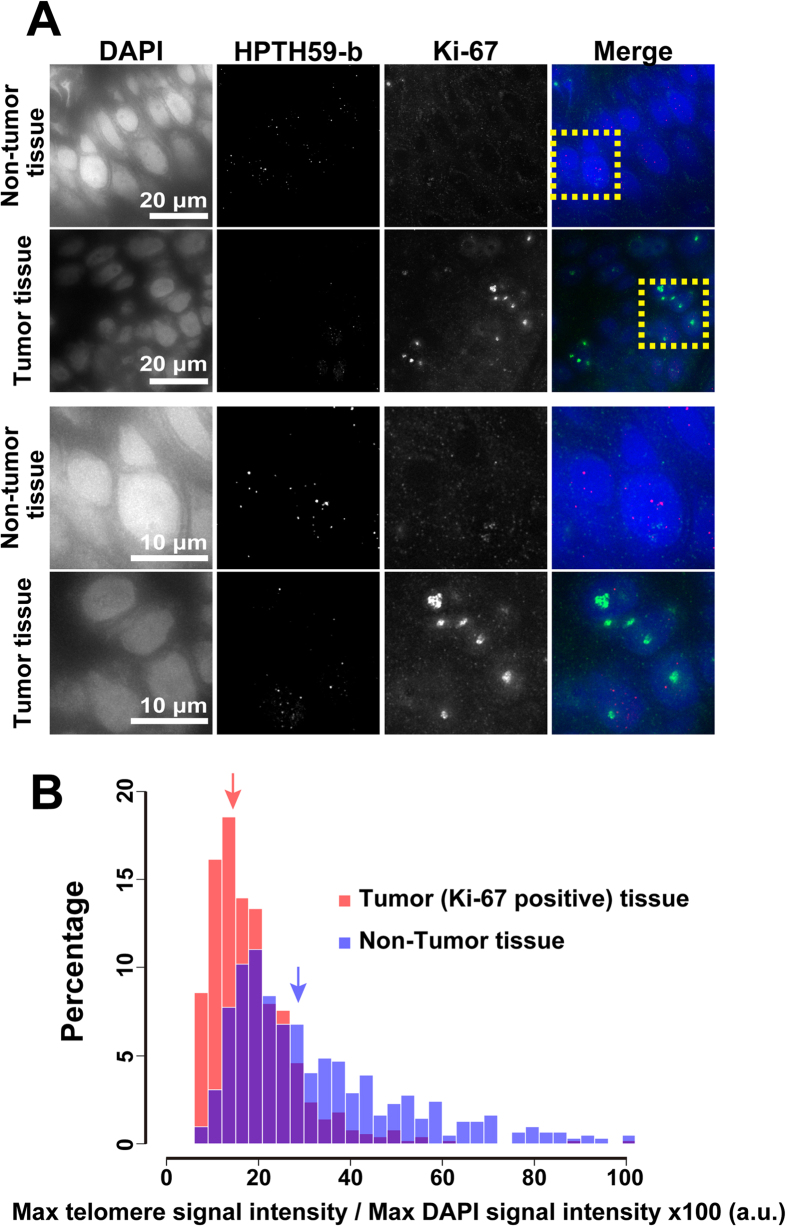
Different telomere lengths between human tumor and non-tumor tissue sections. (**A**) Frozen sections of esophageal tumor/non-tumor tissue stained with DAPI (blue), anti-Ki-67 (growth marker; green) antibody, and HPTH59-b (red). Enlarged images of the boxed region in the upper images are shown in the lower part. (**B**) Distribution histograms of telomere signal intensities in tumor (shown in red; 511 dots from 54 cells) and non-tumor (in blue; 635 dots from 50 cells) tissue sections. Median values of the signal intensities in non-tumor and tumor tissues are 27.8 (blue arrow) and 16.7 (red arrow), respectively. The overlapping area of the two distributions is shown in purple. To compare these median values, the Wilcoxon rank sum test was used (P < 0.01).

Telomere length is strongly connected to cell immortalization and tumorigenesis. Indeed, telomere shortening has been observed in carcinoma from bladder, esophageal, gastric, head and neck, ovarian, and renal cells^36^,^37^. To further investigate telomeres in human neoplastic lesions, we performed simultaneous labeling in esophageal cancer tissue using HPTH59-b and an antibody for a proliferation marker, Ki-67 protein. Ki-67 is highly expressed in cells that are actively dividing, but it is absent in cells under a quiescent state, such as the G0 phase of the cell cycle^38^. We found that some cell fractions in the lesions were still Ki-67-positive (green) and the fluorescent intensity of telomere foci appeared lower than that of Ki-67-negative cells (Fig. 4A). Using digital image analysis, we quantitatively compared telomere lengths (Fig. 4B). The distribution map of the telomere signals revealed two distinct peaks between tumor and non-tumor tissues. These results suggest that highly proliferating tumor cells have shorter telomeres than normal cells, and HPTH59-b can detect differences in telomere length between tumor and non-tumor cells in tissues.

## Discussion

To demonstrate and verify the application of PI polyamide to clinical studies, we used PI polyamide HPTH59-b, along with immunostaining, to visualize telomeres in mouse and human frozen tissue sections. Our quantitative analysis of the telomere signals also suggested that highly proliferating cells in tumor tissue had shorter telomeres than normal cells in non-tumor tissue. Although the issue of whether telomere shortening is a consequence of cell proliferation during tumor expansion or a cause of tumorigenesis initiation is controversial, HPTH59-b provides a simple and quick detection method for telomere alteration of the cancer genome at the single-cell level, thereby contributing to the genetic diagnosis of malignancy and drug response in patients.

Telomere visualization by HPTH59-b has several advantages over the Q-FISH method. First, the sample preparation process is much faster. Even with tissue sections, incubation with HPTH59-b for just 1 hour is sufficient for staining telomeres. Moreover, even when co-staining with HPTH59-b and an antibody for cell-specific markers, the whole procedure can be completed within 4.5 h. The second advantage is high sensitivity. As shown in Supplementary Fig. S2, HPTH59-b generated more telomere signals with lower background noise than did immunostaining with a TRF1 antibody, whereas with cultured cells, HPTH59-b showed a staining efficiency comparable to that of a TRF1 antibody. In the case of thicker tissue sections, the probe size might be critical to accessing telomeres, and HPTH59-b (2.67 kDa) is much smaller than antibodies (~150 kDa). The third advantage is that HPTH59-b labeling can be carried out under mild conditions. This probe can also detect telomere sequences in small DNA molecules in the cytoplasm of ALT cells, which have extra-chromosomal circular DNA of telomeric repeats due to the high activity of recombination^39^,^40^. Only a few studies using the conventional FISH method have ever reported the extra-chromosomal DNA in cytoplasm^32^. This is probably because, unlike large genomic DNA, such small circular plasmid-like DNAs may be easily washed away during the harsh hybridization process and/or easily re-annealed, preventing the nucleic acid probes from hybridization. In contrast, HPTH59-b binds to dsDNA TTAGGG repeats without denaturation, and it may have a similar labeling efficiency for the extra-chromosomal and chromosomal telomere repeats.

Two new telomere labeling methods that use genome editing systems have recently been established. One involves telomere labeling using a transcription activator-like effector (TALE)-based strategy in both fixed and living mammalian cells^41^,^42^. Although the TALE-based method is applicable to live-cell imaging, it is time consuming (i.e., for plasmid construction and establishment of stable cell lines expressing the fluorescent-TALE). Moreover, the TALE-based strategy cannot be applied to telomere labeling in tissue sections, especially clinical samples from patients. On the other hand, HPTH59-b can label telomeres in both cultured cells and tissue sections without have to construct plasmids or establish stable cell lines.

Another recent approach uses the clustered regularly interspaced short palindromic repeats (CRISPR)/CRISPR-associated caspase 9 (Cas9) technique^43^. Recently, Deng *et al*. reported the use of *in vitro* constituted nuclease-deficient CRISPR/Cas9 complexes as probes (Cas9-mediated fluorescence *in situ* hybridization, CASFISH)^44^. Telomere labeling by this method does not require DNA denaturation and can quickly (15 min) label telomeres in cultured cells and tissue sections. On the other hand, the production cost of large amounts of single-guide RNAs (sgRNAs) and dCas9 (nuclease-deficient) protein make it more expensive than the HPTH59-b method, which could be problematic for high-throughput applications, such as those involved in cancer diagnosis.

With the recent development of new technologies, our understanding of chromatin structure and dynamics is deepening^45^. Because our sensitive telomere labeling method can be performed under mild conditions, another interesting application to telomere regions involves super-resolution imaging without harsh treatments. This technique could help to elucidate how telomere chromatin is organized in the cell nuclei. Therefore, telomere visualization using the PI polyamide-based approach discussed here would expand telomere biology and related medical science.

## Methods

### Synthesis

HPTH59-b was synthesized as reported previously^25^.

### Human tissues

The use of human tissues was approved by the committees of the National Center for Global Health and Medicine (#NCGM-G-001766-00) and was in accordance with the Declaration of Helsinki of the World Medical Association. Participants provided written informed consent. We analyzed one case of a patient who had a surgical operation at the National Center for Global Health and Medicine Hospital. Tissues were prepared from regions diagnosed as esophageal squamous cell carcinoma and adjacent normal tissue according to the manufacturer’s protocol. Briefly, excised tissue was flash frozen in cold acetone with optimal cutting temperature (OCT) compound (Sakura Finetek Japan). Tissue sections (10 μm) were prepared by microtome and placed on the slide glass for telomere staining.

### Telomere staining of HeLaS3, HeLa1.3, and U2-OS cells with HPTH59-b

HeLa cells were maintained at 37 °C under 5% CO_2_ atmosphere in DMEM containing 10% fetal bovine serum (FBS). For polyamide staining, cells were grown on coverslips coated with poly-lysine. The cell coverslips were washed in phosphate-buffered saline (PBS) twice and fixed with 1.85% formaldehyde in PBS. The fixed cells on coverslips were stained with HPTH59-b and then mounted as described previously^24^. Section images were recorded with a DeltaVision microscope and deconvolved to eliminate out-of-focus blur to obtain clearer pictures. The deconvolved images were projected (‘Quick Projection’ tool) to obtain the maximum intensity of telomere signals. Non-deconvolved pictures were used for quantitative analysis of HPTH59-b signals.

### Telomere staining of mouse tissue sections with HPTH59-b

Postnatal 0 (P0) mice were fixed in 4% paraformaldehyde in PBS overnight at 4 °C and placed in 30% sucrose in PBS for 1 day at 4 °C. Mouse whole bodies were then embedded in optimal cutting temperature (OCT) compound /30% sucrose (2:1) and incubated for 1 h at room temperature. Embedded samples were stored at −80 °C until sectioning. Samples were sectioned (20 μm) using a CM3050S cryostat (Leica) and kept at −30 °C until use. All experimental protocols were approved by the Animal Committee of the National Institute of Genetics and carried out according to the guidelines to minimize the pain and discomfort of the animals.

### Preparation of human tumor/non-tumor tissue sections

Before staining, sections were incubated in HEN buffer (10 mM HEPES pH 7.5, 1 mM EDTA, 100 mM NaCl) overnight at 37 °C. The sections were permeabilized with 0.1% Triton X-100 for 10 min and briefly washed twice with TEN (10 mM Tris-HCl pH 7.5, 1 mM EDTA, 100 mM NaCl). For blocking, the sections were treated with Normal Goat Serum (NGS) in TE buffer (10 mM Tris-HCl pH 7.5, 1 mM EDTA) for 30 min at room temperature. After a brief rinse with TE buffer, the sections were incubated with 10% NGS, 15 nM HPTH59-b, and 0.5 μg/mL DAPI for 2 h at 37 °C. After washing five times for 3 min with TEN200 buffer (10 mM Tris-HCl pH 7.5, 1 mM EDTA, and 200 mM NaCl), the sections were mounted and image acquisition was performed as described above.

### Co-staining with polyamide and antibody

The procedure from sample preparation to blocking was the same as that described above. The sections were incubated with 10% NGS, 15 nM HPTH59-b, 0.5 μg/mL DAPI, and either anti-DDX4/MVH antibody (Abcam #ab13840, 1:500 dilution) or anti-Ki-67 antibody (Oncogene #NA59, 1:20 dilution) for 2 h at 37 °C^46^, following the process from washing to image acquisition as described above.

### Quantification of telomere signals

Images of HeLa1.3 and HeLaS3 cells and human/mouse tissue sections were recorded with a DeltaVision microscope under identical conditions. Non-deconvolved images were projected and used as source images. Extraction of each telomere signal was performed as follows. The background fluorescent signals of HPTH59-b and DAPI in the source images were subtracted using the ‘Rolling ball’ tool (Fiji)^47^. By setting an arbitrary threshold value of HPTH59-b intensity, the telomere spots were contoured. The maximal signal intensities of HPTH59-b and DAPI were extracted from each telomere spot, and each HPTH59-b signal was then normalized with DAPI signals. The Wilcoxon rank sum test was used for statistical analysis, which was performed with R software^48^.

## Additional Information

**How to cite this article**: Sasaki, A. *et al*. Telomere Visualization in Tissue Sections using Pyrrole-Imidazole Polyamide Probes. *Sci. Rep.* **6**, 29261; doi: 10.1038/srep29261 (2016).

## Supplementary Material

Supplementary Information

## Figures and Tables

**Figure 1:**
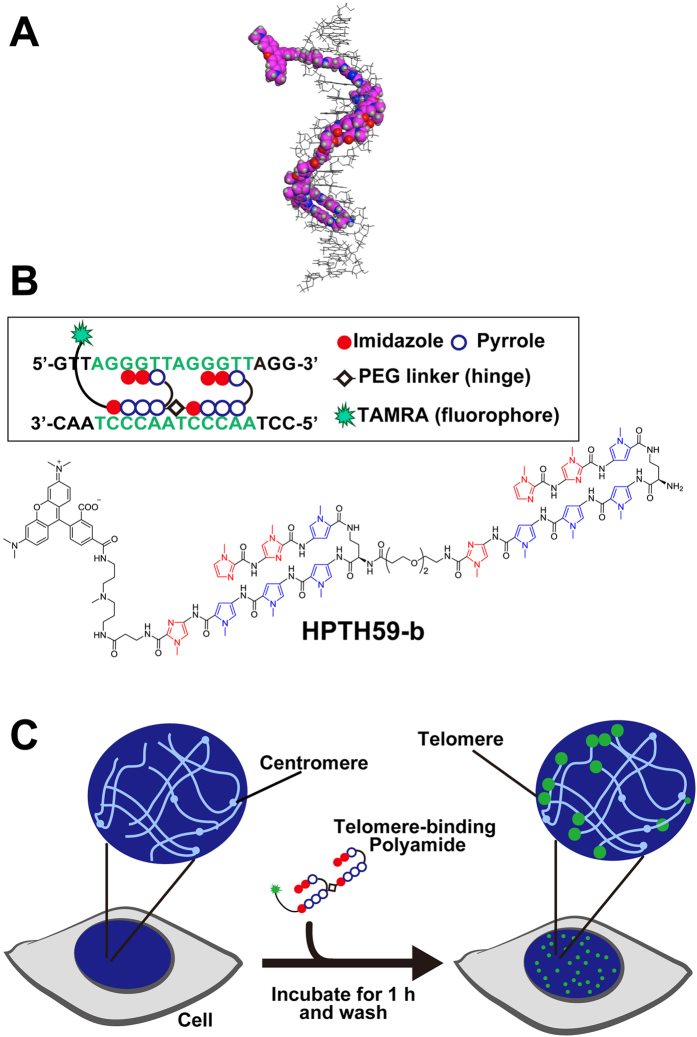
Structure of fluorescent polyamide HPTH59-b and scheme for telomere labeling. (**A**) A structural model of HPTH59-b binding to DNA. (**B**) Chemical structure of HPTH59-b. TAMRA is tetramethylrhodamine. In the box, the base recognition profile of HPTH59-b is shown. (**C**) A simple scheme for telomere labeling using HPTH59-b. Green dots indicate signals from telomere repeats.

## References

[b1] Blackburn E. H. Telomeres and telomerase: the means to the end (Nobel lecture). Angew Chem Int Ed 49, 7405–7421 (2010). 10.1002/anie.201002387 20821774

[b2] Zakian V. A. Telomeres: the beginnings and ends of eukaryotic chromosomes. Exp Cell Res 318, 1456–1460 (2012). 22391099 10.1016/j.yexcr.2012.02.015 PMC3372703

[b3] Smogorzewska A. & de Lange T. Regulation of telomerase by telomeric proteins. Annu Rev Biochem 73, 177–208 (2004). 15189140 10.1146/annurev.biochem.73.071403.160049

[b4] Shay J. W. & Bacchetti S. A survey of telomerase activity in human cancer. Eur J Cancer 33, 787–791 (1997). 9282118 10.1016/S0959-8049(97)00062-2

[b5] Kim N. W. . Specific association of human telomerase activity with immortal cells and cancer. Science 266, 2011–2015 (1994). 7605428 10.1126/science.7605428

[b6] Bryan T. M., Englezou A., Gupta J., Bacchetti S. & Reddel R. R. Telomere elongation in immortal human cells without detectable telomerase activity. Embo J 14, 4240–4248 (1995). 7556065 10.1002/j.1460-2075.1995.tb00098.x PMC394507

[b7] Bryan T. M., Englezou A., Dalla-Pozza L., Dunham M. A. & Reddel R. R. Evidence for an alternative mechanism for maintaining telomere length in human tumors and tumor-derived cell lines. Nat Med 3, 1271–1274 (1997). 9359704 10.1038/nm1197-1271

[b8] Lansdorp P. M. . Heterogeneity in telomere length of human chromosomes. Hum Mol Genet 5, 685–691 (1996). 8733138 10.1093/hmg/5.5.685

[b9] Henderson S., Allsopp R., Spector D., Wang S. S. & Harley C. **In situ** analysis of changes in telomere size during replicative aging and cell transformation. J Cell Biol 134, 1–12 (1996). 8698806 10.1083/jcb.134.1.1 PMC2120915

[b10] Levsky J. M. & Singer R. H. Fluorescence *in situ* hybridization: past, present and future. J Cell Sci 116, 2833–2838 (2003). 12808017 10.1242/jcs.00633

[b11] Meeker A. K. . Telomere length assessment in human archival tissues: combined telomere fluorescence *in situ* hybridization and immunostaining. Am J Pathol 160, 1259–1268 (2002). 11943711 10.1016/S0002-9440(10)62553-9 PMC1867217

[b12] Meeker A. K. . Telomere shortening is an early somatic DNA alteration in human prostate tumorigenesis. Cancer Res 62, 6405–6409 (2002). 12438224

[b13] Ferlicot S. . Measurement of telomere length on tissue sections using quantitative fluorescence *in situ* hybridization (Q-FISH). J Pathol 200, 661–666 (2003). 12898604 10.1002/path.1392

[b14] Meeker A. K. . Telomere shortening occurs in subsets of normal breast epithelium as well as *in situ* and invasive carcinoma. Am J Pathol 164, 925–935 (2004). 14982846 10.1016/S0002-9440(10)63180-X PMC1614707

[b15] Trauger J. W., Baird E. E. & Dervan P. B. Recognition of DNA by designed ligands at subnanomolar concentrations. Nature 382, 559–561 (1996). 8700233 10.1038/382559a0

[b16] White S., Szewczyk J. W., Turner J. M., Baird E. E. & Dervan P. B. Recognition of the four Watson-Crick base pairs in the DNA minor groove by synthetic ligands. Nature 391, 468–471 (1998). 9461213 10.1038/35106

[b17] Chenoweth D. M. & Dervan P. B. Allosteric modulation of DNA by small molecules. Proc Natl Acad Sci USA 106, 13175–13179 (2009). 19666554 10.1073/pnas.0906532106 PMC2726366

[b18] Dervan P. B. Molecular recognition of DNA by small molecules. Bioorg Med Chem 9, 2215–2235 (2001). 11553460 10.1016/s0968-0896(01)00262-0

[b19] Dervan P. B. & Edelson B. S. Recognition of the DNA minor groove by pyrrole-imidazole polyamides. Curr Opin Struct Biol 13, 284–299 (2003). 12831879 10.1016/s0959-440x(03)00081-2

[b20] Dervan P. B., Doss R. M. & Marques M. A. Programmable DNA binding oligomers for control of transcription. Curr Med Chem Anticancer Agents 5, 373–387 (2005). 16101489 10.2174/1568011054222346

[b21] Bando T. & Sugiyama H. Synthesis and biological properties of sequence-specific DNA-alkylating pyrrole-imidazole polyamides. Acc Chem Res 39, 935–944 (2006). 17176032 10.1021/ar030287f

[b22] Blackledge M. S. & Melander C. Programmable DNA-binding small molecules. Bioorg Med Chem 21, 6101–6114 (2013). 23665141 10.1016/j.bmc.2013.04.023 PMC3789866

[b23] Maeshima K., Janssen S. & Laemmli U. K. Specific targeting of insect and vertebrate telomeres with pyrrole and imidazole polyamides. Embo J 20, 3218–3228 (2001). 11406598 10.1093/emboj/20.12.3218 PMC150199

[b24] Kawamoto Y. . Development of a new method for synthesis of tandem hairpin pyrrole-imidazole polyamide probes targeting human telomeres. J Am Chem Soc 135, 16468–16477 (2013). 24083880 10.1021/ja406737n

[b25] Hirata A. . Structural evaluation of tandem hairpin pyrrole-imidazole polyamides recognizing human telomeres. J Am Chem Soc 136, 11546–11554 (2014). 25036716 10.1021/ja506058e

[b26] Kawamoto Y. . Tandem trimer pyrrole–imidazole polyamide probes targeting 18 base pairs in human telomere sequences. Chem Sci 6, 2307–2312 (2015). 10.1039/c4sc03755c PMC5645774 29308145

[b27] Hastie N. D. . Telomere reduction in human colorectal carcinoma and with ageing. Nature 346, 866–868 (1990). 2392154 10.1038/346866a0

[b28] Bryan T. M., Englezou A., Dunham M. A. & Reddel R. R. Telomere length dynamics in telomerase-positive immortal human cell populations. Exp Cell Res 239, 370–378 (1998). 9521855 10.1006/excr.1997.3907

[b29] Sommerfeld H. J. . Telomerase activity: a prevalent marker of malignant human prostate tissue. Cancer Res 56, 218–222 (1996). 8548767

[b30] Takai K. K., Hooper S., Blackwood S., Gandhi R. & de Lange T. *In vivo* stoichiometry of shelterin components. J Biol Chem 285, 1457–1467 (2010). 19864690 10.1074/jbc.M109.038026 PMC2801271

[b31] Scheel C. . Alternative lengthening of telomeres is associated with chromosomal instability in osteosarcomas. Oncogene 20, 3835–3844 (2001). 11439347 10.1038/sj.onc.1204493

[b32] Tokutake Y. . Extra-chromosomal telomere repeat DNA in telomerase-negative immortalized cell lines. Biochem Biophys Res Commun 247, 765–772 (1998). 9647768 10.1006/bbrc.1998.8876

[b33] Fujiwara Y. . Isolation of a DEAD-family protein gene that encodes a murine homolog of Drosophila vasa and its specific expression in germ cell lineage. Proc Natl Acad Sci USA 91, 12258–12262 (1994). 7991615 10.1073/pnas.91.25.12258 PMC45416

[b34] Toyooka Y. . Expression and intracellular localization of mouse Vasa-homologue protein during germ cell development. Mech Dev 93, 139–149 (2000). 10781947 10.1016/s0925-4773(00)00283-5

[b35] Yoshioka H., McCarrey J. R. & Yamazaki Y. Dynamic nuclear organization of constitutive heterochromatin during fetal male germ cell development in mice. Biol Reprod 80, 804–812 (2009). 19129513 10.1095/biolreprod.108.072603 PMC2804833

[b36] Wentzensen I. M., Mirabello L., Pfeiffer R. M. & Savage S. A. The association of telomere length and cancer: a meta-analysis. Cancer Epidemiol Biomarkers Prev 20, 1238–1250 (2011). 21467229 10.1158/1055-9965.EPI-11-0005 PMC3111877

[b37] Zhang C. . The Association between Telomere Length and Cancer Prognosis: Evidence from a Meta-Analysis. PLoS One 10, e0133174 (2015). 26177192 10.1371/journal.pone.0133174 PMC4503690

[b38] Schluter C. . The cell proliferation-associated antigen of antibody Ki-67: a very large, ubiquitous nuclear protein with numerous repeated elements, representing a new kind of cell cycle-maintaining proteins. J Cell Biol 123, 513–522 (1993). 8227122 10.1083/jcb.123.3.513 PMC2200129

[b39] Dunham M. A., Neumann A. A., Fasching C. L. & Reddel R. R. Telomere maintenance by recombination in human cells. Nat Genet 26, 447–450 (2000). 11101843 10.1038/82586

[b40] Nabetani A. & Ishikawa F. Alternative lengthening of telomeres pathway: recombination-mediated telomere maintenance mechanism in human cells. J Biochem 149, 5–14 (2011). 20937668 10.1093/jb/mvq119

[b41] Ma H., Reyes-Gutierrez P. & Pederson T. Visualization of repetitive DNA sequences in human chromosomes with transcription activator-like effectors. Proc Natl Acad Sci USA 110, 21048–21053 (2013). 24324157 10.1073/pnas.1319097110 PMC3876203

[b42] Miyanari Y., Ziegler-Birling C. & Torres-Padilla M. E. Live visualization of chromatin dynamics with fluorescent TALEs. Nat Struct Mol Biol 20, 1321–1324 (2013). 24096363 10.1038/nsmb.2680

[b43] Chen B. . Dynamic imaging of genomic loci in living human cells by an optimized CRISPR/Cas system. Cell 155, 1479–1491 (2013). 24360272 10.1016/j.cell.2013.12.001 PMC3918502

[b44] Deng W., Shi X., Tjian R., Lionnet T. & Singer R. H. CASFISH: CRISPR/Cas9-mediated *in situ* labeling of genomic loci in fixed cells. Proc Natl Acad Sci USA 112, 11870–11875 (2015). 26324940 10.1073/pnas.1515692112 PMC4586837

[b45] Maeshima K., Ide S., Hibino K. & Sasai M. Liquid-like behavior of chromatin. Curr Opin Genet Dev 37, 36–45 (2016). 26826680 10.1016/j.gde.2015.11.006

[b46] Maeshima K. . Cell-cycle-dependent dynamics of nuclear pores: pore-free islands and lamins. J Cell Sci 119, 4442–4451 (2006). 17074834 10.1242/jcs.03207

[b47] Schneider C. A., Rasband W. S. & Eliceiri K. W. NIH Image to ImageJ: 25 years of image analysis. Nat Methods 9, 671–675 (2012). 22930834 10.1038/nmeth.2089 PMC5554542

[b48] Core Team R. (2013). R: A language and environment for statistical computing. R Foundation for Statistical Computing, Vienna, Austria.

