# Supplementary material for: Telomere Visualization in Tissue Sections using Pyrrole–Imidazole Polyamide Probes
Source: Sci Rep. 2016 Jul 6;6:29261. doi: 10.1038/srep29261 (PMC4933941; doi:10.1038/srep29261)
Supplement: Supplementary Information [file srep29261-s1.pdf]

Supplementary Information

Telomere Visualization in Tissue Sections using Pyrrole–Imidazole Polyamide Probes

Asuka Sasaki, Satoru Ide, Yusuke Kawamoto, Toshikazu Bando, Yukinori Murata, Mari Shimura, Kazuhiko Yamada, Akiyoshi Hirata, Kiyoshi Nokihara, Tatsumi Hirata, Hiroshi Sugiyama, Kazuhiro Maeshima

Supplementary Methods

Telomere staining of HeLa1.3 and HeLaS3 chromosome spreads with HPTH59-b. The chromosome spreads were prepared and stained as described previously.[26] The quantitative analysis of the telomere signals was performed as described in Methods.

Co-staining of mouse tissue sections with anti TRF1 antibody and HPTH59-b. Section preparation was performed as described in Methods. The sections were incubated with 10% NGS, 1000-fold diluted rabbit anti-TRF1 antibody, 15 nM HPTH59-b, and 0.5 μg/mL DAPI for 2 h at 37°C. The following process was carried out as described in Methods. Colocalization of HPTH59-b and TRF1 signals were analyzed using the ‘RG2B colocalization’ plugin in Fiji

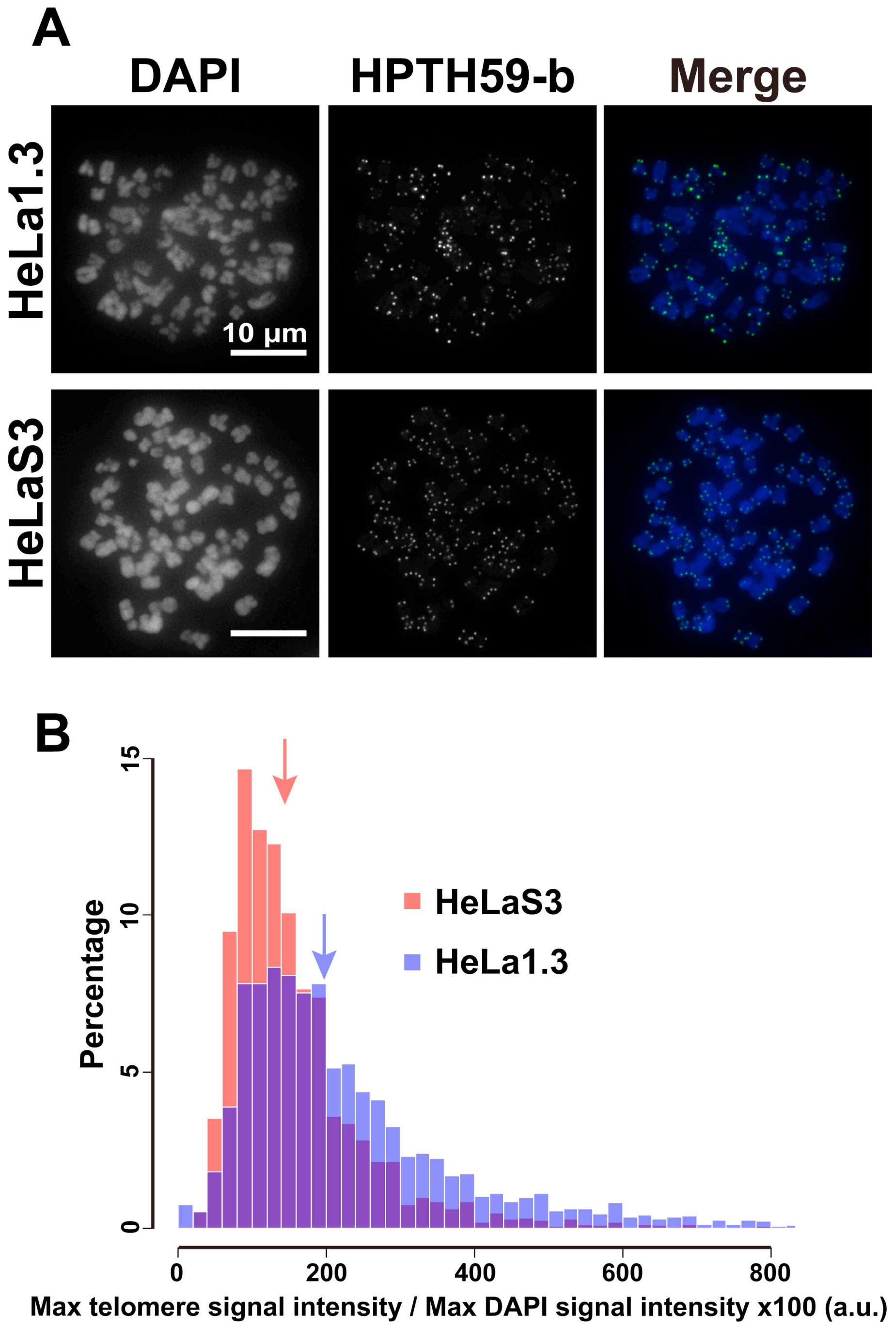

Supplementary Figure S1

Telomere labeling in chromosomal spreads with HPTH59-b. (A) HeLa1.3 (1st row) and HeLaS3 (2nd row) cell spreads stained with DAPI (1st column) and HPTH59-b (2nd column). The 3rd column is the merged images. **(B)** Distribution histograms of telomere signal intensities in HeLa1.3 (in blue; 6564 dots from 38 cells) and HeLaS3 (in red; 10895 dots from 43 cells) cell spreads. Median values of signal intensities in non-tumor and tumor tissues are 194 (blue arrow) and 135 (red arrow), respectively. The overlapping area of the two distributions is shown in purple. To compare these median values, the Wilcoxon rank sum test was used ($P<0.01$).

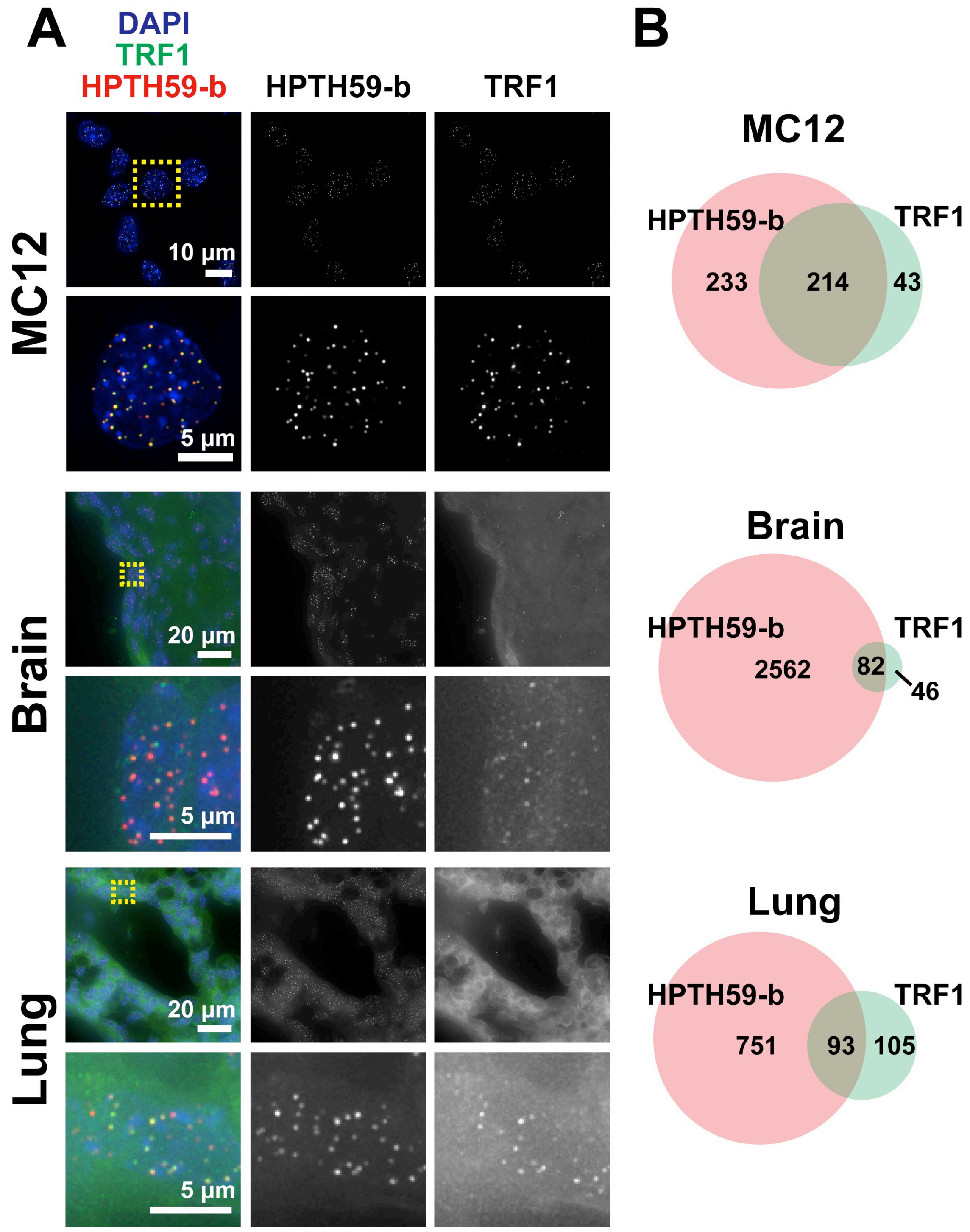

Supplementary Figure S2

Co-staining with HPTH59-b and TRF-1 antibody in cultured cells and tissue sections. (A) Mouse embryonic carcinoma cells (MC12) and frozen mouse tissue sections stained with DAPI (blue), HPTH59-b (red) and TRF1 antibody (green). Enlarged images of the boxed region in the upper row are shown in the lower row. **(B)** Venn diagrams of telomere signals derived from HPTH59-b and TRF1. The number of dots was extracted from images in (A).

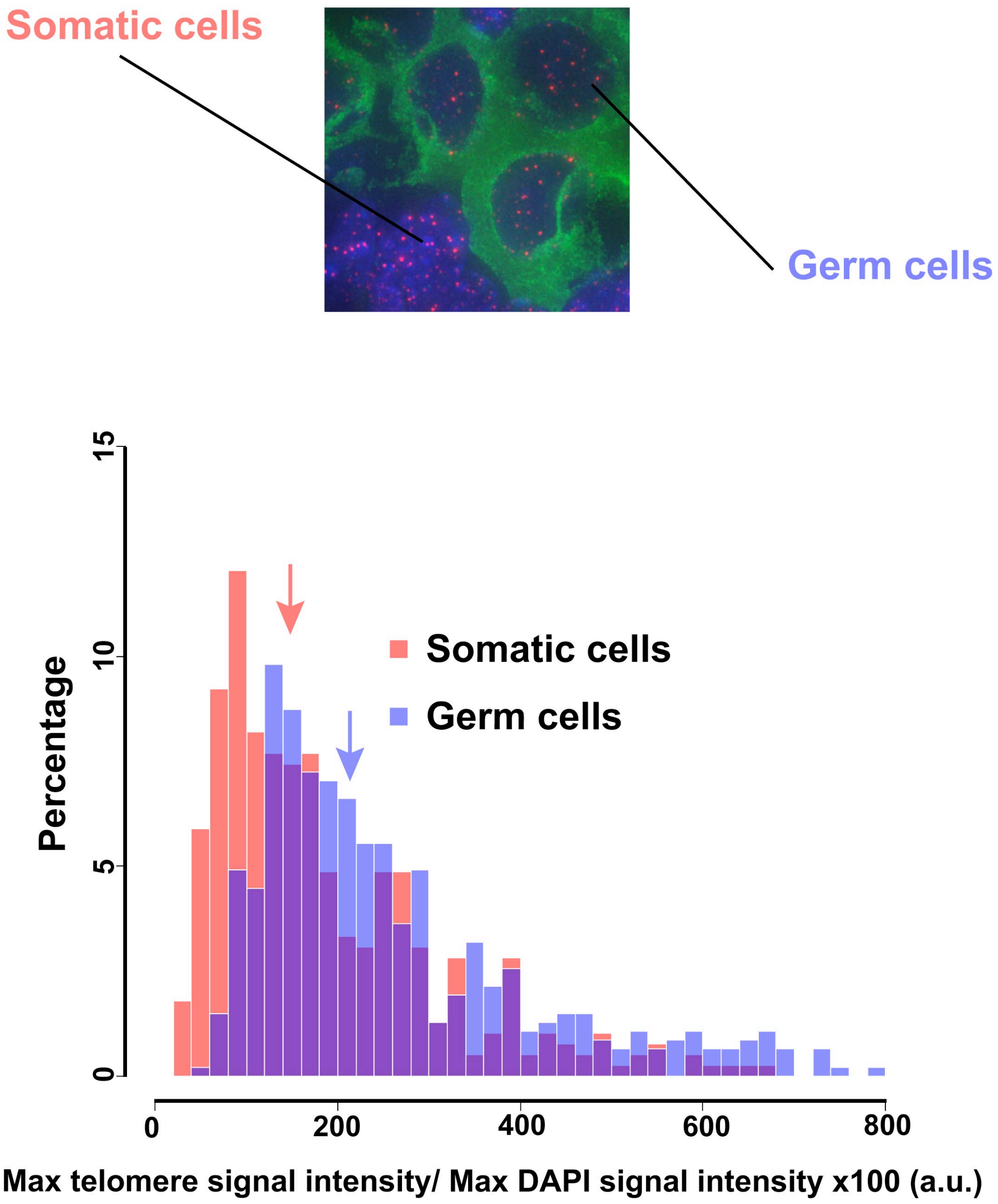

Supplementary Figure S3

Distribution histograms of telomere signal intensities in germ cells and somatic cells. We analyzed 19 cells (469 dots) and 19 cells (390 dots) in germ (blue) and somatic (red) cells, respectively. The identical image containing PGCs in Fig. 3B is shown at the top. Median values of signal intensities in germ and somatic cells are 220 (blue arrow) and 152 (red arrow), respectively. The overlapping area of the two distributions is shown in purple. To compare these median values, the Wilcoxon rank sum test was used ($P<0.01$).
